# A Method of Solubilizing and Concentrating Astaxanthin and Other Carotenoids

**DOI:** 10.3390/md19080462

**Published:** 2021-08-16

**Authors:** Kiyotaka Y. Hara, Shuwa Yagi, Yoko Hirono-Hara, Hiroshi Kikukawa

**Affiliations:** 1 Laboratory of Environmental Bioengineering, Graduate Division of Nutritional and Environmental Sciences, University of Shizuoka, 52-1 Yada, Suruga-ku, Shizuoka 422-8526, Japan; yc383537@bk2.so-net.ne.jp (S.Y.); kikukawa@u-shizuoka-ken.ac.jp (H.K.); 2 Department of Environmental and Life Sciences, School of Food and Nutritional Sciences, University of Shizuoka, 52-1 Yada, Suruga-ku, Shizuoka 422-8526, Japan; y-hirono@u-shizuoka-ken.ac.jp

**Keywords:** astaxanthin, marine carotenoid, crustacyanin, European lobster, NusA-tag, *Escherichia coli*

## Abstract

The valuable marine carotenoid, astaxanthin, is used in supplements, medicines and cosmetics. In this study, crustacyanin, an astaxanthin-binding protein, was used to solubilize and concentrate astaxanthin. The recombinant crustacyanin of European lobster spontaneously formed an inclusion body when it was over-expressed in *Escherichia coli*. In this study, fusing the NusA-tag to the crustacyanin subunits made it possible to express in a soluble fraction and solubilize astaxanthin in aqueous solution. By cutting off the NusA-tag, the crustacyanin subunits generated the pure insoluble form, and captured and concentrated astaxanthin. Overall, the attaching and releasing NusA-tag method has the potential to supply solubilized carotenoids in aqueous solution and concentrated carotenoids, respectively.

## 1. Introduction

Carotenoids are used as red or orange pigments for foods, supplements, pharmaceuticals and cosmetics. Carotenoids are found as natural compounds in microorganisms, algae, plants and animals [1,2,3,4,5,6]. A valuable carotenoid, astaxanthin (3,3′-dihydroxy-β,β-carotene-4,4′-dione; C_40_H_52_O_4_), acts as a protective agent against oxidative damage to cells in vivo because it has high antioxidant activity [7,8,9]. Astaxanthin for human consumption is often produced by extraction from the microalgae, *Haematococcus pluvialis* [10]; generally extracted from *H. pluvialis* using super-critical CO_2_, a method which is not harmful with easy removal of the CO_2_ [11,12]. However, the efficiency of extracting astaxanthin from *H. pluvialis* cells has been reported to be less than 50%, even when carried out under high pressure (30 MPa) [13,14]. Repeating the super-critical extraction of astaxanthin several times also leads to very high running costs [15,16,17,18]. In addition, astaxanthin is generally extracted with organic solvents and can be concentrated by evaporation [19]. For a sustainable society in the future, environmentally-negative solvent discharge and energy-intensive evaporation processes are not desirable. Therefore, there is a need to develop an environmentally friendly astaxanthin concentrating method. Furthermore, providing astaxanthin in the form of an aqueous solution is important for developing applications of this valuable product, such as cosmetics and nutritional supplement drinks.

In the cells of the shells of crustaceans, astaxanthin exists with its binding protein, crustacyanin. Wald et al. first extracted crustacyanin from the shells of lobsters [20]. Crustacyanin subunits can be classified into two groups according to the composition of their amino acids, molecular size and peptide mapping. One group consists of the A1, C1 and C2 subunits, and the other consists of the A2 and A3 subunits [21]. Heterologous expression of the crustacyanin subunits in *E. coli* has also been carried out to clarify their three-dimensional structures [22]. One possible method of concentrating carotenoids is to use astaxanthin-binding proteins. However, the expression levels of these crustacyanin subunits are lower than those required for its application. 

The current study has developed a simple method for solubilizing and concentrating astaxanthin using an easily-purified single crustacyanin protein. Using the attaching and releasing NusA-tag method, the crustacyanin protein was solubilized with the NusA-tag, followed by cleavage of the tag, which easily induces a pure precipitate for concentrating astaxanthin. It has been demonstrated that the method developed in this study can be used not only for astaxanthin, the original substrate of crustacyanin, but also for α-carotene and β-carotene. 

## 2. Results and Discussion

### 2.1. Optimization of Crustacyanin Preparation

**Figure 1:**
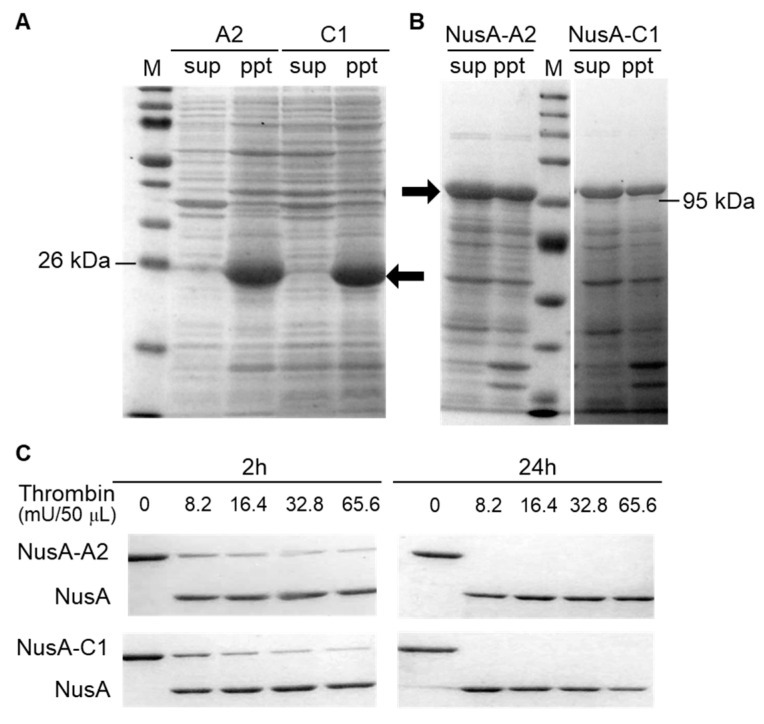
Expression and digestion pattern of crustacyanin. (**A**) Native crustacyanin A2 and C1 are represented by the arrows; (**B**) NusA-tagged crustacyanin A2 and C1 proteins are represented by the arrows. The abbreviations “sup”, “ppt” and “M” represent supernatant, pellet and molecular marker, respectively; (**C**) Cleaving the NusA-tag from the A2 and C1 proteins tagged with NusA. Biotinylated thrombin was added to NusA-A2 and NusA-C1 crustacyanin in a 50 μL reaction mixture at different concentrations shown for 2 or 24 h. Concentration of acrylamide used in SDS-PAGE is 8.0%.

For the first preparation step, native European lobster crustacyanin was produced in *E. coli*. The expression levels of A2 and C1 were higher than those of the other subunits [21], and A2C1 was a main subcomplex which forms *H. gammarus* crustacyanin [22]. Therefore, genes artificially synthesized with codon optimization for *E. coli* which encode the native *H. gammarus* crustacyanin A2 and C1 proteins were introduced into *E. coli*. Figure 1A shows that almost all the A2 (23 kDa) and C1 (26 kDa) proteins were expressed as insoluble inclusion bodies in *E. coli*. Therefore, their expressions in the inclusion bodies made the application of these proteins difficult. The NusA-tag is a protein known to increase the solubility of its conjugated target protein. In the present study, the NusA-tag (molecular weight calculated as 55 kDa and shown as 69 kDa in SDS-PAGE) [23] was fused to the N-terminal of the A2 and C1 proteins. As a result, the A2 and C1 proteins tagged with NusA were expressed in the soluble fraction at almost the same level as that of the inclusion bodies (Figure 1B). 

In the second step, to construct a simple method to purify the crustacyanin proteins, the NusA-tag was removed from the NusA-tagged crustacyanin proteins. The protease, thrombin, was used to digest the spacer region between the NusA-tag and the crustacyanin proteins. To optimize the conditions for digestion, the NusA-A2 and NusA-C1 proteins (0.2 μg/mL) were digested using the biotinylated thrombin at different concentrations (0, 8.2, 16.4, 32.8 and 65.6 mU) at 4 °C for different reaction times (2, 4, 8 or 24 h). After this digestion reaction in the presence of 65.6 mU thrombin protease for 2 h, both the NusA-A2 and NusA-C1 proteins were almost completely digested to generate the A2 and C1 proteins (Figure 1C). In contrast, a reaction time of 24 h using less than 8.2 mU thrombin protease was sufficient for the complete digestion of NusA from the NusA-A2 and NusA-C1 proteins.

### 2.2. Carotenoid Recovery by Insoluble Crustacyanin

**Figure 2:**
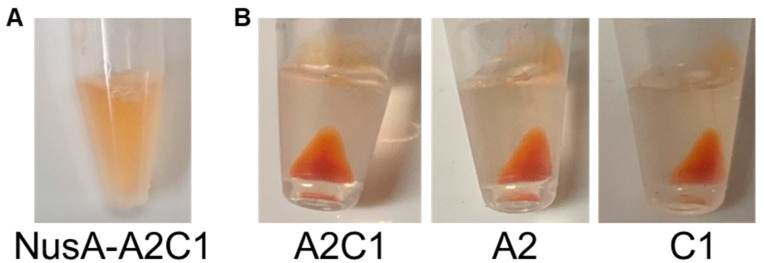
Solubilization and concentration of astaxanthin by crustacyanin subunits. (**A**) Addition of astaxanthin to crustacyanin A2 and C1 subunits attaching the NusA-tag; (**B**) Addition of astaxanthin to crustacyanin A2C1, A2 and C1 after digesting the NusA-tag.

The crustacyanin A2C1 complex has been reported to have the ability to bind to astaxanthin [21]. The binding ability of A2C1 was compared when tagged with or without NusA. The NusA-A2C1 and NusA-free A2C1 complexes were constructed by mixing the NusA-A2/NusA-C1 proteins and NusA-free A2/C1 proteins, respectively. The astaxanthin was solubilized in buffer with the NusA-A2C1 complex and no precipitation was observed after centrifugation (Figure 2), which indicated that the astaxanthin had solubilized in the aqueous phase.

This method of solubilizing astaxanthin in aqueous solution could be applied in various industries, particularly for cosmetics and supplemental nutrition drinks. In contrast, the digestion of the NusA-tag from the NusA-A2 and NusA-C1 proteins and NusA-A2C1 complex constructed from the resulting NusA-free proteins generated a red-colored precipitate after binding to astaxanthin and centrifugation (Figure 2). These precipitates indicate the ability of the method to capture and concentrate astaxanthin of a high purity as the precipitated form of crustacyanin. 

**Figure 3:**
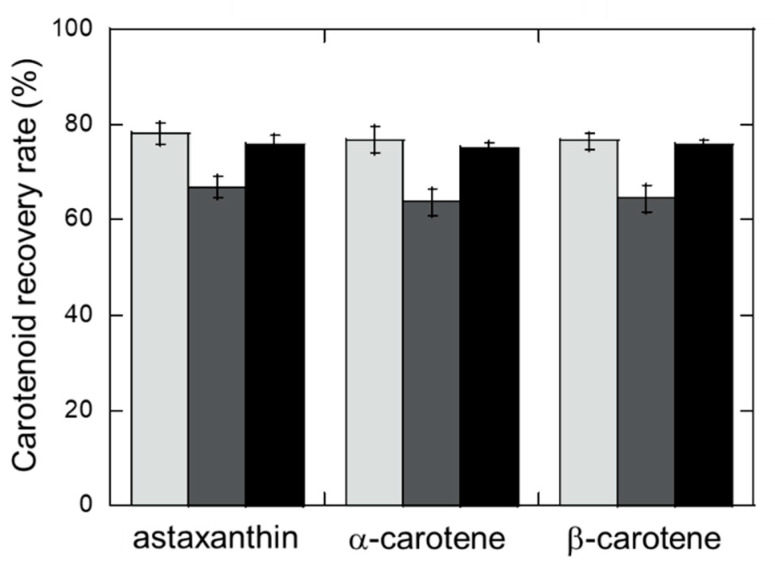
Capturing carotenoids from a mixture using the crustacyanin subunits. The crustacyanin subunits were reacted with a mixture of carotenoids containing astaxanthin, α-carotene and β-carotene, each at a concentration of 20 μg/mL. The amount of each carotenoid captured in the crustacyanin was measured by high-performance liquid chromatography and their recovery rates were calculated from the amounts added. The light gray, dark gray and black bars represent the crustacyanin A2, C1 subunits and A2C1 complex, respectively. Error bars represent the standard deviations.

To evaluate whether the crustacyanin subunits could be used to concentrate carotenoids other than astaxanthin, the abilities of the A2 or C1 protein and their A2C1 complex to bind α-carotene and β-carotene were compared (Figure 3). The crustacyanin A2 protein individually bound all three carotenoids at a recovery rate (>75%) higher than that for the C1 protein, and almost equal to that for the A2C1 complex. These results indicated that the expression of both the A2 and C1 proteins and the formation of their complex is not required for the efficient capture of carotenoids. The crustacyanin A2 subunit alone provided a simple method for concentrating carotenoids. Figure 3 also shows no differences in the recovery rate for astaxanthin, α-carotene and β-carotene from the mixture. This wide-ranging ability to capture carotenoids indicates the potential of this method for application to any other type of carotenoid.

## 3. Materials and Methods

### 3.1. Strains, Plasmids and Transformation

Genes encoding the European lobster *Homarus gammarus* crustacyanin A2 and C1 subunits (Gene bank accession No. LC598211 and No. LC598212, respectively) were artificially synthesized with codon optimization for *Escherichia coli* (Eurofins Genomic, Tokyo, Japan). These genes were amplified using PCR, then subcloned into the pET-21d vector (Merck KGaA, Darmstadt, Germany) to construct pET21d-A2 (the forward primer and reverse primer used in this amplification were 5′-GTGCGGCCGCAAGCTTCGCGCGATACACGCATTCG-3′ and 5′-CGACAAGAGTCCGGGAGCTCTGGACGGCATTCCGTCCTTTG-3′, respectively) and pET21d-C1 (the forward primer and reverse primer used in this amplification were 5′-GTGCGGCCGCAAGCTTCAGCGTTTTCTGGGTATCATACGG-3′ and 5′-CGACAAGAGTCCGGGAGCTCTGGACAAGATTCCCGATTTTGTTG-3′, respectively). *E. coli* Rosetta2 (Merck KGaA) was then transformed using pET21d-A2 or pET21d-C1 to obtain *E. coli* strains which could over-express native A2 or C1 subunits induced by IPTG. The PCR fragments including the A2 or C1 genes were also subcloned into the pET43.1a vector (Merck KGaA) to construct pET43.1a-A2 and pET43.1a-C1. *E. coli* Origami B (Merck KGaA) was transformed by pET43.1a-A2 or pET43.1a-C1 to obtain *E. coli* strains which could over-express the A2 or C1 subunits with the NusA-tag (Nus⋅Tag) at their N-terminus. These *E. coli* Origami B mutant strains were then transformed using the pTF16 vector (Takara Bio, Shiga, Japan), which contains the gene-encoding Tig chaperone induced by L-arabinose, to construct *E. coli* Origami B (pET-43.1a-A2/pTF-16) and Origami B (pET-43.1a-C1/pTF-16).

### 3.2. Preparation of Soluble NusA-Crustacyanin

The *E. coli* Rosetta 2 and Origami B mutant strains were cultured in LB medium (10 g/L tryptone, 5 g/L yeast extract and 5 g/L sodium chloride) supplemented with 0.5 mg/mL L-arabinose and 100 μg/mL ampicillin in an Erlenmeyer flask, stirred at 100 rpm at 37 °C. When the cell concentration reached an OD_600_ value of 0.5, 1 mM IPTG was added and then cultured at 100 rpm for a further 3 h at 30 °C. After centrifugation (3000× *g*, 5 min, 4 °C), the supernatant was removed and the cell pellet was suspended in an appropriate volume of 50 mM sodium phosphate buffer (pH 7.0), then sonicated for a total of 45 s (on 5 s/off 5 s × 9 cycles) using an ultrasonic disruptor (Vibra Cell, Sonics & Materials Inc, Newtown, CT, USA). After centrifugation (1060× *g*, 5 min, 4 °C), ammonium sulfate (30% saturated concentration) was rapidly added to the disrupted cell extract, then incubated for 30 min at 4 °C. Fifteen milliliters of the resulting solution was centrifuged (1060× *g*, 5 min, 4 °C), then the supernatant was removed. The remaining pellet was resuspended in 3 mL of 50 mM sodium phosphate buffer (pH 7.0), dialyzed in the same buffer overnight, followed by replacement with 3 mL of fresh buffer and additional dialysis for 4 h. The dialyzed NusA-A2 and NusA-C1 proteins were centrifuged (1060× *g*, 5 min, 4 °C), then the concentration of each solubilized protein in the supernatant was measured using a Protein Assay BCA Kit (FUJIFILM Wako Pure Chemical Corp., Osaka, Japan). Equal volumes of the NusA-A2 and NusA-C1 solutions were mixed to form a NusA-A2C1 complex as described previously [22]. The chemicals were purchased from Nacalai Tesque (Kyoto, Japan) or FUJIFILM Wako Pure Chemical Corp.

### 3.3. Preparation of NusA-free Crustacyanin

NusA was digested from the NusA-A2 or NusA-C1 protein as described in the manual of the Novagen^®^ Thrombin kits (Merk KGaA, Darmstadt, Germany). The biotinylated thrombin was used at different concentrations at 4 °C for different reaction times. Ten microliters of the reaction mixture were applied to SDS-PAGE to check the efficiency of digesting NusA from the NusA-A2 and NusA-C1 proteins. Under the optimized digestion conditions, large amounts (0.4 mg/mL) of NusA-A2 and NusA-C1 proteins were separately digested with 320 mU biotinylated thrombin at 4 °C for 3 days to prepare enough amounts of NusA-free crustacyanin for the next experiment described in Section 3.4. The NusA-free A2 and C1 proteins were collected through precipitation by centrifugation (1060× *g*, 5 min, 4 °C) and removing the supernatant. The proteins were concentrated by resuspension in the appropriate volume of 50 mM sodium phosphate buffer (pH 7.0).

### 3.4. Concentrating Carotenoids Using NusA-free Crustacyanin

Sixty microliters of a carotenoid mixture containing 20 μg/mL each of astaxanthin, α-carotene and β-carotene were dissolved in acetone, then gently mixed with 0.1 mL of each of the single NusA-free A2 or NusA-free C1 proteins (all at a concentration of 1.0 mg/mL) and 50 μL of their A2C1 complex generated from A2 and C1 proteins (at a concentration of 1.0 μg/μL). After centrifugation, the carotenoids precipitated with the A2 or C1 proteins or the A2C1 complex were suspended in 0.4 mL of acetone, vortexed and centrifuged (16,000× *g*, 5 min, 4 °C) to obtain the first supernatant for recovering carotenoids. To recover the remaining carotenoids from each protein, the precipitated protein was resuspended in 0.2 mL of acetone, vortexed and centrifuged (16,000× *g*, 5 min, 4 °C), and then the resulting second supernatant was mixed with the first supernatant (total 0.6 mL). The carotenoid concentrations were determined as described previously [24,25] using a high-performance liquid chromatography system (Shimadzu, Kyoto, Japan) equipped with a COSMOSIL Cholester Packed Column (4.6 i.d. × 250 mm, Nacalai Tesque, Kyoto, Japan). The operating conditions were: column temperature, 35 °C; mobile phase, methanol/tetrahydrofuran (80/20 (*v*/*v*)); flow rate, 1.0 mL/min; and detection of carotenoids at 470 nm using a UV detector (SPD-20AV, Shimadzu).

## 4. Conclusions

In this study, we developed an efficient method for producing crustacyanin subunits and used them to solubilize and concentrate carotenoids. Crustacyanin subunits with a NusA-tag attached to their N-terminus achieved soluble expression in *E. coli* and solubilized astaxanthin in aqueous solution. Astaxanthin could be concentrated by cleaving the NusA-tag from the crustacyanin subunit followed by its precipitation with astaxanthin. Furthermore, this method also could be applied to α-carotene and β-carotene. These results should indicate the potential of the attaching and releasing NusA-tag method to supply a solubilization process by attaching the NusA-tag, and a concentration process by releasing the NusA-tag, for the production of a broad range of carotenoids.

## Data Availability

The article contains all the data produced in this study.

## Abbreviations

IPTG, isopropyl-β-D-thiogalactopyranoside; PCR, polymerase chain reaction; SDS-PAGE, sodium dodecyl sulfate-polyacrylamide gel electrophoresis.
